# Genome-Wide Association Study for Body Length, Body Height, and Total Teat Number in Large White Pigs

**DOI:** 10.3389/fgene.2021.650370

**Published:** 2021-08-02

**Authors:** Yifeng Hong, Jian Ye, Linsong Dong, Yalan Li, Limin Yan, Gengyuan Cai, Dewu Liu, Cheng Tan, Zhenfang Wu

**Affiliations:** ^1^College of Animal Science and National Engineering Research Center for Breeding Swine Industry, South China Agricultural University, Guangzhou, China; ^2^National Engineering Research Center for Breeding Swine Industry, Wens Foodstuff Group Co., Ltd., Yunfu, China

**Keywords:** genome-wide association study, body length, body height, total teat number, Large white pigs

## Abstract

Body length, body height, and total teat number are economically important traits in pig breeding, as these traits are usually associated with the growth, reproductivity, and longevity potential of piglets. Here, we report a genetic analysis of these traits using a population comprising 2,068 Large White pigs. A genotyping-by-sequencing (GBS) approach was used to provide high-density genome-wide SNP discovery and genotyping. Univariate and bivariate animal models were used to estimate heritability and genetic correlations. The results showed that heritability estimates for body length, body height, and total teat number were 0.25 ± 0.04, 0.11 ± 0.03, and 0.22 ± 0.04, respectively. The genetic correlation between body length and body height exhibited a strongly positive correlation (0.63 ± 0.15), while a positive but low genetic correlation was observed between total teat number and body length. Furthermore, we used two different genome-wide association study (GWAS) approaches: single-locus GWAS and weighted single-step GWAS (WssGWAS), to identify candidate genes for these traits. Single-locus GWAS detected 76, 13, and 29 significant single-nucleotide polymorphisms (SNPs) associated with body length, body height, and total teat number. Notably, the most significant SNP (S17_15781294), which is located 20 kb downstream of the *BMP2* gene, explained 9.09% of the genetic variance for body length traits, and it also explained 9.57% of the genetic variance for body height traits. In addition, another significant SNP (S7_97595973), which is located in the *ABCD4* gene, explained 8.92% of the genetic variance for total teat number traits. GWAS results for these traits identified some candidate genomic regions, such as SSC6: 14.96–15.02 Mb, SSC7: 97.18–98.18 Mb, SSC14: 128.29–131.15 Mb, SSC17: 15.39–17.27 Mb, and SSC17: 22.04–24.15 Mb, providing a starting point for further inheritance research. Most quantitative trait loci were detected by single-locus GWAS and WssGWAS. These findings reveal the complexity of the genetic mechanism of the three traits and provide guidance for subsequent genetic improvement through genome selection.

## Introduction

External characteristics such as body length, body height, and total teat number are very important in the pig industry. Body length and body height are not directly considered as pig breeding target traits. However, body length and other morphological characteristics often affect the value of the animal when purchasing a sow. Besides, the growth potential of pigs has a negative effect on the longevity of sows (Hong et al., 2019). A large body length, flat rib shape, narrow body width, and upright leg posture might be adverse features for sow longevity (Nikkila et al., 2013). An increase in the number of offspring leads to the need for more nipples; therefore, selecting the total teat number is an effective strategy to improve the reproductive performance of sows (Rohrer and Nonneman, 2017). Genome-wide association studies (GWAS) have been widely used to analyze the genetic architecture of reproduction, meat quality, and production traits in the pig population (Verardo et al., 2016; Schmid et al., 2018; Ding et al., 2019). Previous GWAS found that several single nucleotide polymorphisms (SNPs) in or near the bone morphogenetic protein 2 (*BMP2*) gene were associated with body length and body height, and many reported that the vertebrae development-associated (*VRTN*) gene was significantly associated with total teat number in pigs (Lee et al., 2014, 2019; Arakawa et al., 2015; Wang et al., 2017). Almost all GWAS for body length, body height, and total teat number were performed by single-locus GWAS. Weighted single-step GWAS (WssGWAS) is widely used in association analysis to increase the number of genotyped animals in livestock (Suwannasing et al., 2018; Lee et al., 2019; Fu et al., 2020). WssGWAS is a promising method allowing utilization of phenotypes from ungenotyped animals based on pedigree information, so we compared the result from two methods on body length, body height, and total teat number in pigs. On one hand, we can obtain more candidate genes. On the other hand, we can verify the reliability of GWAS result based on two methods.

High-density genetic markers can identify linkage disequilibrium (LD) in large populations (Marchini et al., 2007). In many scenarios, although there is a downtrend in sequencing cost, it is still expensive to use whole-genome sequencing in agricultural breeding. By simplifying genome sequencing, the cost of genotyping animals can be greatly reduced, further enabling the application of genome-wide selection (Wen et al., 2017). Imputation-based strategies are key to simplifying genome sequencing, because it increases the density of genetic markers (Howie et al., 2012). The accuracy of imputation influences the results of the analysis.

As one of the most commonly used pig breeds, Large White pigs are often used as the standard in modern commercial settings. This breed has the characteristics of large litters, heavy milk production, and strong maternal instincts. It can be used in a crossbreeding program in different ways, such as terminal sire on rare breeds, maternal sire in Landrace breeds, and commercial sire. Large White pigs are meek and less fat, which is in line with market demand^1^.

In this study, we used genotyping-by-sequencing (GBS) technology to genotype Large White pigs and analyzed the genetic correlation between body length, body height, and total teat number. We combined single-locus and weighted single-step GWAS for these traits in the Large White population.

## Materials and Methods

### Ethics Statement

All procedures related to animals in this study were performed strictly in line with the guidelines for the care and use of experimental animals established by the Ministry of Science and Technology of the People’s Republic of China (Approval Number: 2006-398). All the scheme design experiments using animals were approved by the ethics committee of South China Agricultural University (SCAU, Guangzhou, China). The experimental animals were not anesthetized or euthanized for the purpose of this study.

### Sample Collection and Phenotyping

The Large White sows used for this study were born between January 2012 and November 2016. All 2,068 sows were subjected to the same growth and feeding conditions and raised in a single nuclear farm in Wen’s Foodstuff Group (Guangdong, China). The following phenotypic data were used in this study: body length, body height, and total teat number. Body length was measured from the base of the pig’s ears to the base of its tail when the weight of the pigs reached 100 ± 5 kg. Body height was measured from the shoulders to the ground when the pig’s weight reached 100 ± 5 kg. Tapes were used to measure body length and height. The total teat number in this study was the sum of normal left and right teats and was recorded within 48 h after birth.

### DNA Extraction, Genotyping, and Quality Control

Genomic DNA was extracted from the ear tissue using the QIAamp DNA Mini kit (QIAGEN, Hilden, Germany) and checked using agarose gel electrophoresis. The genomic DNA concentration was determined by a NanoDrop 2000 spectrophotometer (Thermo Fisher Scientific, Waltham, MA, United States), and then diluted to 40 ng/μL in 96-well plates. Genome-wide SNP discovery and genotyping was performed using GBS technology. DNA samples (160 ng) were digested with two restriction endonucleases *EcoR*I and *Msp*I, then ligated to the digested adapters. Following adapter ligation, samples were pooled in 96-plex and size-selected using two cycles of purification with Agencourt AMPure XP Beads (Beckman Coulter, Pasadena, CA, United States). The Agilent 2100 Bioanalyzer was used to assess the library inserts’ fragment size. The qualified libraries were sequenced on the Illumina Hiseq platform by 150-bp paired-end sequencing. Raw reads were pre-processed using Trimmomatic (version 0.33) to remove the adapter sequences and low quality reads. SNP genotyping were called according to the pipeline implemented in TASSEL 5.0 with default parameters (Bradbury et al., 2007), and Beagle 5.1 (Browning et al., 2018) was used to impute missing SNP genotypes. SNP quality control was conducted by Plink (version 1.07) with the parameters of Hardy-Weinberg equilibrium *P* > 10–6, minor allele frequency >0.01, individuals call rate >0.95, and SNPs call rate >0.99 (Purcell et al., 2007). Finally, a total of 195,895 SNP markers passed quality control in all 2,068 Large White pigs.

### Principal Components Analysis and Linkage Disequilibrium Analysis

Principal component analysis (PCA) was performed using GCTA software (Yang et al., 2011). LD analysis was performed with PopLDdency software (Zhang et al., 2019). The average LD decay distance (r2 = 0.2) across the whole genome and each chromosome genome was analyzed.

### Genetic Parameters Estimation

Genetic parameters were estimated using AIREMLK90 in BLUPF90 software packages (Legarra et al., 2014). A univariate animal model was used to calculate heritability, and a bivariate animal model was used to calculate genetic correlation. The models used were as follows:   Y  =  Xb  +  Za  +  Wt  +  e, where Y is the vector of phenotypic records, b is the vector of fixed effects (including year-season), a is the vector of additive genetic variation, t is the vector of litter effect, e is the vector of residuals, and X, Z, and W are incidence matrices for b, a, and e, respectively.

### Single-Locus GWAS

In the current study, association tests with univariate linear mixed models were performed using GEMMA software (Zhou and Stephens, 2012, 2014). The statistical model was as follows: Y  =  Zβ   +  Wa  +  u  +  e, where Y is the vector of phenotypic records, β is the vector of the regression coefficients, including the intercept, a is the effect size of the marker, u is the vector of random effects, e is the vector of random errors, Z is a matrix of covariates (fixed effect such as year-season), W is the vector of SNP genotypes (u∼MVN_*n*_(0,λτ^–1^K),e ∼MVN_*n*_(0,τ^–1^I_*n*_), where λ is the ratio between the two variance components, K is a known relatedness matrix, In is the identity matrix, and MVNn denotes the dimensional multivariate normal distribution). Considering that the Bonferroni correction is a stringent criterion, we used Plink (version 1.07) to generate a refined subset of SNPs that were in approximate linkage equilibrium with each other. The genome-wide significant threshold was set as 0.05/N, and the chromosome-wide significant threshold was set as 1/N, where N is the number of the independent SNPs. To further confirm whether the signal in the candidate region was caused by the top SNP, conditional analysis was performed by adding the genotypes of these variants in the model as fixed effects.

### WssGWAS

WssGWAS was performed using the programs implemented in the BLUPF90 family. Firstly, RENUMF90, which is a renumbering program, was run to create an input file for BLUPF90 programs and provide basic statistics. Then, we ran BLUPF90 with genomic information and saved A22^–1^ and G^–1^, where A22 is a numerator relationship matrix for genotyped animals, and G is a genomic relationship matrix. Finally, we ran POSTGSF90 to obtain the effect of SNP on the additive variance explained by segments. The results were presented in terms of the additive genetic variance contribution explained by each window of 50 SNPs.

### Search for Genes and Functional Enrichment Analysis

In the single-locus GWAS method, we searched for candidate genes that were the nearest to the SNPs that reached a potentially significant level according to their physical position in the genome, as published on the EnsEMBL website^2^. In the GWAS method, several peaks explaining >0.5% of the direct additive genetic variance were discovered for these traits. Functional enrichment analysis was performed using the Gene Ontology, Kyoto Encyclopedia of Genes and Genomes, and UniProt databases. Furthermore, a functional enrichment analysis was performed using Metascape^3^ (Zhou et al., 2019), which is based on a *Homo sapiens* (human) database and using the default parameters.

## Results

### Summary of Phenotype, Genotype, and Genetic Parameter Statistics

The descriptive statistics of body length, body height, and total teat number for the Large White sows are described in Table 1. Briefly, the average value for body length, body height, and total teat numbers were 121.11 ± 3.55, 61.41 ± 2.57, and 13.88 ± 1.04, respectively. The heritability of body length, body height, and total teat number were 0.25 ± 0.04, 0.11 ± 0.03, and 0.22 ± 0.04, respectively. The genetic correlation and phenotypic correlation of body length, body height, and total teat number for the Large White sows were shown in Table 2. The phenotypic and genetic correlations between body length and body height were high and positive, which were 0.47 ± 0.02 and 0.63 ± 0.15, respectively. There was a positive but low genetic correlation between body length and total teat number. However, a very low genetic correlation was observed between total teat number and body height. Genetic correlation among these traits showed that selected body length traits also indirectly selected body height traits, and selected total teat number traits did not influence the body length and body height traits in pigs.

**TABLE 1:** Summary statistics of three traits in Large White pig population.

Trait^1^	Number of records	Mean ( ± SD)^2^	^3^Min	^4^Max	^5^C.V.	^6^H^2^ (±SE)
BL	2068	121.11 ± 3.55	110	133	2.93	0.25 ± 0.04
BH	1581	61.41 ± 2.57	54	70	4.18	0.11 ± 0.03
TTN	2068	13.88 ± 1.04	7	18	7.49	0.22 ± 0.04

*^1^BL = body length, BH = body height, TTN = total teat number.*

*^2^Mean = mean value, SD = standard deviation.*

*^3^Min = minimum value.*

*^4^Max = maximum value.*

*^5^C.V. = coefficient of variation.*

*^6^H^2^ = heritability value.*

**TABLE 2:** Genetic correlation and phenotypic correlation of three traits in Large White pig population.

Trait^1^	BL	BH	TTN
BL		0.47 ± 0.01	0.01 ± 0.01
BH	0.63 ± 0.15		−0.01 ± 0.01
TTN	0.13 ± 0.12	−0.08 ± 0.20	

*^1^BL = body length; BH = body height; TTN = total teat number; estimation of genetic correlation below the diagonal and phenotypic correlation above the diagonal.*

### Genetic Architecture and LD Decay

The PCA plot is shown in Supplementary Figure 1B. The figure shows that our population did not have population stratification. The average LD decay distance was different among the chromosomes, and the fastest and the slowest LD decay occurred in SSC8 and 13, respectively. The average LD decay distance was approximately 300 kb, where the r2 values fell to 0.20 (Supplementary Figure 1A).

### Single-Locus GWAS for Three Traits

**FIGURE 1:**
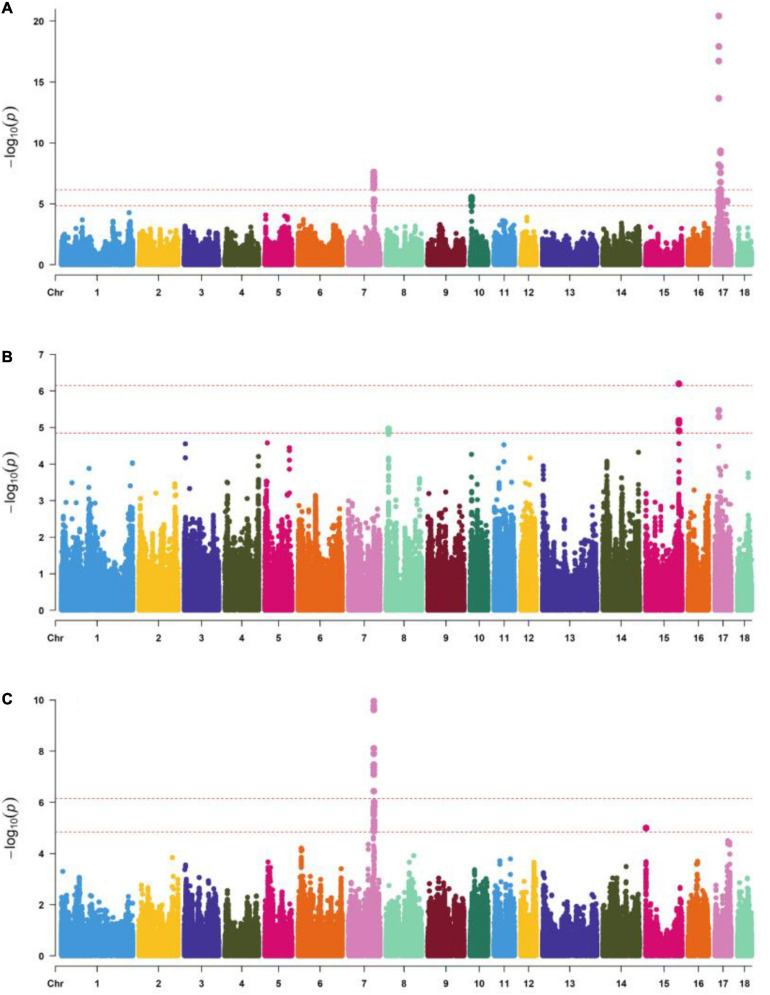
Manhattan plots of genome-wide SNP significance for **(A)** body length, **(B)** body height, and **(C)** total number teats. The *X* axis shows SNPs across chromosomes SSC1 to SSC18, and the *Y* axis represents the –log10(P). The top line indicates the genome-wide significant thresholds (*P* < 7.03E-7) and the bottom line indicates the suggestive genome-wide significant thresholds (*P* < 1.41E-5).

The number of independent SNPs was 71,220, the genome-wide significant threshold and chromosome-wide significant threshold were equal to 7.03E-7 and 1.41E-5, respectively, Significant SNPs (*P* < 1.41E-5) were detected for body length, body height, and total teat number, and are shown in Supplementary Table 1 and Figure 1. A total of 40, 1, and 13 SNPs reached extremely significant levels in body length, body height, and total teat number traits (*P* = 7.03E-7). Besides, 76, 13, and 29 SNPs reached a suggestive significance level (*P* = 1.41E-5). In terms of body length traits, 27 SNPs on SSC7, 3 SNPs on SSC15, and 46 SNPs on SSC17 exceeded the chromosome-wide significance level (*P* = 1.41E-5). The lowest *P*-value of SNPs was 3.89E-21, located on SSC17. In the body height traits, there were 4, 6, and 3 SNPs that exceeded the chromosome-wide significance level (*P* = 1.41E-5) on SSC8, SSC15, and SSC17, respectively. In the total teat number, 28 SNPs were located on SSC7, and 1 SNP located on SSC15 exceeded the threshold (*P* = 1.41E-5). The SNP with the lowest *P*-value (*P* = 1.10E-10) was S7_97595973 on SSC7. Significantly, a total of 24 SNPs that exceeded the chromosome-wide significance level (*P* = 1.41E-5) were all exhibited in the traits of body length and total teat number.

### WssGWAS for Three Traits

**FIGURE 2:**
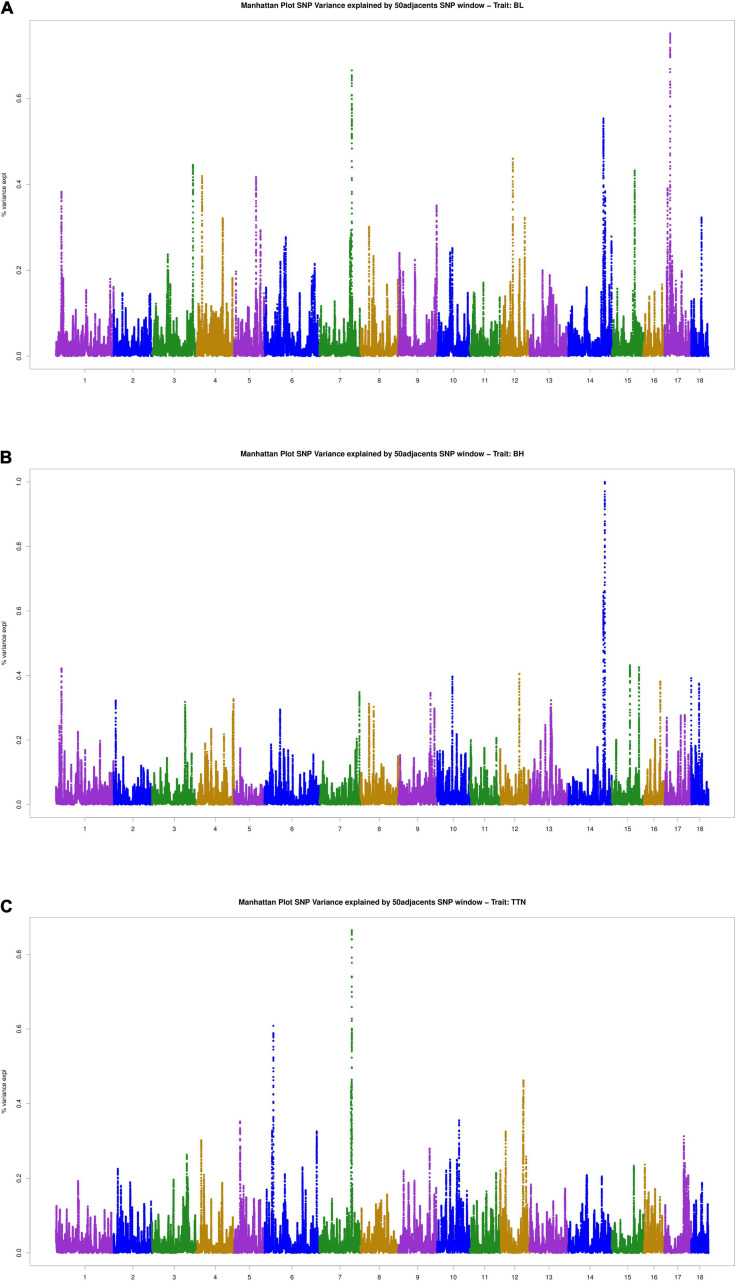
Manhattan plots for the percentage of genetic variance by 50 adjacent SNP windows for **(A)** body length, **(B)** body height, and **(C)** total teat number. Variance expl (%) represents the proportion of genetic variance explained by 50 adjacent SNPs.

**FIGURE 3:**
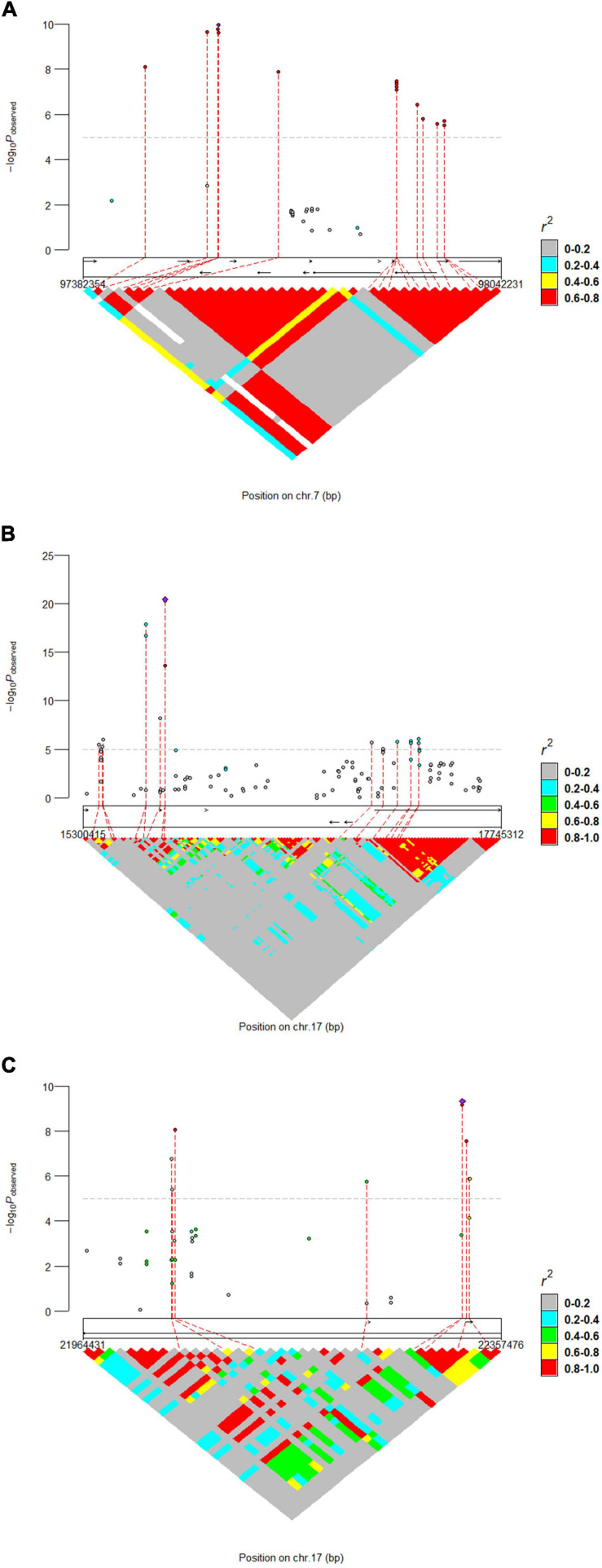
Locus-specific Manhattan plot and linkage disequilibrium analysis for **(A)** total teat number and **(B,C)** body length. The horizontal dashed line indicates the significance thresholds (*P*-value = 1.41E-5). The most significant SNP within each region was annotated with corresponding candidate genes.

The results of WssGWAS for body length, body height, and total teat number were grouped by the proportion of genetic variance, explained by windows of 50 successive SNPs in Figure 2. The genomic windows that explained more than 0.5% of the additive genetic variance are shown in Supplementary Table 2. For the body length trait, three genomic windows that were located on SSC7, 14, and 17 were detected. For the body height trait, a series of successive genomic windows of a 3 Mb region in SSC14 was detected. For the trait of the total teat number, two genomic windows were discovered in SSC6 and SSC7. Importantly, a region (SSC7: 97.18–97.87 Mb) explains more than 0.5% of the additive genetic variance relevant to the traits of body length and total teat number. In addition, the genetic windows at SSC14: 128.29–128.86 Mb were also associated with body length and height traits. The results of single-locus GWAS and WssGWAS showed the same quantitative trait loci (QTL) region at the traits of body length and total teat number, and almost significant SNPs were mapped to different LD blocks in the region of SSC7: 97.38–98.04 Mb (Figure 3). We also analyzed the LD pattern of the significant SNPs for the body length trait in SSC17: 15.30–17.75 Mb and SSC17: 21.96–22.36 Mb.

### Search for Candidate Genes and Functional Enrichment Analysis

The result of single-locus GWAS in the body length trait discovered an SNP associated with the *BMP2* gene, located on the 15.75 Mb of SSC17. In WssGWAS, the most significant SNPs related to the *PLCB1* gene were discovered at position 17.05 Mb on SSC17. In addition, a significant SNP in SSC7 related to the *AREL1* gene was detected at position 97.88 Mb. In the result of the body height trait, we also discovered *BMP2* as a candidate gene, and the most significant SNP was discovered at position 127.85 Mb on SSC15, near the ENSSSCG00000043546 gene. In WssGWAS, we discovered a region of approximately 2.86 Mb on SSC14, which contained approximately 23 candidate genes, and the region explained 1% genetic variance. For the total teat number trait, the most significant SNPs were located in the *ABCD4* gene on SSC7. In WssGWAS, we also discovered that this region was associated with the total teat number trait. However, the most significant SNP was related to the *ENTPD5* and *COQ6* genes. After combining the SNP sets from the two GWAS results, a total of 35, 20, and 27 functional genes were identified for body length, body height, and total teat number, respectively. The result of GO analysis showed that these genes were enriched in 10 functional categories, including fat cell differentiation, cofactor metabolic process, behavior, cilium organization, regulation of intracellular transport, negative regulation of phosphorylation, dephosphorylation, developmental process involved in reproduction, cofactor metabolic process, and small molecule catabolic process (*P* < 0.01, Supplementary Table 3).

## Discussion

### Population Structure and Genetic Parameters Statistics

In this study, we used GBS technology to obtain 195,895 high-quality SNPs. Despite the genomic selection (GS) and GWAS methods, the advantage of GBS has been proven in previous studies (Meuwissen and Goddard, 2010; Elshire et al., 2011). GBS can obtain more recombinant fragments than SNP chips and provide high SNP coverage at a low cost. Population stratification is the main factor that influences the accuracy of GWAS, and a great number of the Large White pigs had no population stratification which supports our result. The average LD decay was very fast, it may be that the Large White population was strongly selected. We used these SNPs to calculate genetic parameters for body length, body height, and total teat number traits. The heritability of body length and total teat number was 0.25 ± 0.04 and 0.22 ± 0.04, respectively. Other studies have reported that the heritability of body length and total teat number was 0.16 to 0.32 and 0.19 to 0.365, respectively (Johnson and Nugent, 2003; Tan et al., 2017; Zhuang et al., 2020). The heritability of body height was lower than that reported in other literature (Zhou et al., 2019). The genetic correlation between body length and body height was 0.63 ± 0.15, there was a strong positive genetic correlation between body length and body height.

### Candidates Regions and Genes

In both single-locus GWAS and WssGWAS, many SNPs found to be located on SSC7 between the 97.18 and 99.07 Mb regions reached a suggestive significance level for body length and total teat number traits. Though similar, these QTLs are not identical copies of one another (Supplementary Table 1). The most significant SNPs of these two traits were located in different genes. One was located upstream of the *VRTN* gene and another was located downstream of the *VRTN* gene. In a previous study, the VRTN gene was associated with carcass length, vertebral number, and teat number (Lopes et al., 2014; Yang et al., 2016; Falker-Gieske et al., 2019). In a recent study, conditional analyses, LD pattern assessment, and the phenotype distribution pattern of the *VRTN* alleles showed that the *VRTN* gene may not be a strong or the only candidate causal gene for teat number (Zhuang et al., 2020). In the trait of total teat number, the most significant SNP was near the *ABCD4* gene, which is expected to have a negative effect on protein function (Zhao et al., 2020). We also performed conditional analyses and found that no signal remained when we used an SNP (S7_97595288) as a fixed effect. Two missense mutations in the *ABCD4* gene were discovered; moreover, both of these two variants and lots of non-coding variants were present in all Landrace wt/wt animals (van Son et al., 2019). Thus, *ABCD4* is more likely to be a causal gene.

In the body length traits, at SSC7, the most significant SNP within *AREL1* was located 250.95 kb downstream of *VRTN*. It may be a high LD with the true causal mutation in this QTL region, causing the *VRTN* to be affected. *BMP2* and *PLCB1* genes were more likely to be true candidate genes in the body length trait. The SNP (S17_15781294) explained 9.09% of the genetic variance and neared the *BMP2* gene. Zhou et al. reported the *BMP2* gene as a candidate gene associated with body length and body height traits in Chinese Shushan pigs (Zhou et al., 2019). Moreover, *BMP2* as a candidate gene was discovered and associated with carcass length in pigs (Falker-Gieske et al., 2019). *BMP2* was also associated with the distal midpiece reflex for sperm traits in a Duroc boar population (Zhao et al., 2020). *BMP2* plays an important role in the regulation of ovarian folliculogenesis and luteal formation in humans (Bai et al., 2020). We also discovered that the SNP (S17_15781304) explained 9.57% genetic variance and neared the *BMP2* gene for body height trait. In the WssGWAS method, the continuous SNP explained that the top genetic variance was located in the *PLCB1* gene. Meng et al. (2017) reported that the *PLCB1* gene contributes to growth at the onset of puberty in a Yorkshire purebred pig population.

In WssGWAS, the QTL region SSC14: 128.29–128.86 Mb was associated with body length and body height traits. This QTL segment has not been reported as a GWAS result for these traits in pigs previously. The *CACUL1* gene was located in this region and has been reported to be associated with anthropometric traits such as height and weight in humans (Galvan-Femenia et al., 2018). Interestingly, the *BMP2* gene was also related to body length and body height in the single-locus GWAS result, and the QTL region on SSC14: 128.29 – 128.86 Mb was also associated with body length and body height. These findings were the same as the WssGWAS result. Both body length and height belonged to growth traits, and there was a high genetic correlation between them. From this, it is easy to understand that some QTLs or QTL regions were found to be associated with body length and body height, and these can also reflect the accuracy of our results. The 122 SNPs associated with body height were located on SSC14 between 128.29 and 131.15 Mb. This was a relatively large region, which contained approximately 20 genes and explained about 1% of the genomic variation. Nevertheless, according to the pig QTL databases, there was no body height-related gene discovered in this region. Although some SNPs showed high LD; it was still difficult to determine which gene significantly affected body height. In this region, the *WDR11* gene has been associated with congenital hypogonadotropic hypogonadism and Kallmann syndrome, which are human developmental genetic disorders defined by delayed puberty and infertility (Kim et al., 2018). A search for *GRK5* as a putative candidate determined that it is associated with residual feed intake in pigs (Do et al., 2014).

In another candidate gene, *CPEB2* is necessary for proper porcine meiotic maturation and embryonic development (Prochazkova et al., 2018). In the WssGWAS method, the SNPs associated with total teat number were located on SSC6: 14.96 – 15.02 Mb. This region contains *DHODH*, *ENSSSCG00000002749*, *TXNL4B*, *DHX3B*, and *PMFBP1* genes. Mutations in *PMFBP1* cause acephalic spermatozoa syndrome that leads to infertility in male mice (Zhu et al., 2018; Li et al., 2019).

### Comparison Using Two Methods

In our study, we used two methods to perform GWAS. Compared with single-locus GWAS, WssGWAS has the following advantages. Firstly, it can analyze individuals with phenotypes and pedigrees but no genotyped data (Zhang et al., 2016). Secondly, it can be adapted to complex animal models, such as the repeatability model. Wu et al. (2018) used WssGWAS to analyze reproductive traits such as total number born and the number of born alive traits in pigs. Gao et al. (2019) utilized a WssGWAS procedure to detect genetic regions and further candidate genes associated with semen traits in a Duroc boar population. Lastly, WssGWAS can reduce noise and highlight the most significant peaks (Wang et al., 2014). In our study, two approaches were mutually verified and supplemented. WssGWAS can target QTL regions associated with traits, but it is difficult to target some true candidate genes. A small effective population size and a small number of independent chromosome segments could affect the variance of the SNP windows. A few segments explained 1% of the total variance in our study, but it still not enough to determine whether we discovered a significant QTL region. This is likely because we had a large number of SNPs or possibly because of the specific characteristics of this dataset. Here, we reported the chromosome segments that explained more than 0.5% of the additive genetic variance as the significant QTL region. Compared with single-locus GWAS, WssGWAS is a powerful and potentially useful tool, especially when statistical models of analysis are complex.

## Conclusion

In conclusion, we used two methods to perform GWAS for body length, body length, and total teat number traits. GWAS results for three important agricultural traits identified a series of candidate genes and showed their genetic architecture. These findings revealed the complexity of the genetic mechanism of the three traits, providing guidance for subsequent genetic improvement through genome selection.

## Data Availability Statement

The original contributions presented in the study are included in the article/Supplementary Material, further inquiries can be directed to the corresponding authors.

## Ethics Statement

The animal study was reviewed and approved by the Ethics Committee of South China Agricultural University.

## Author Contributions

ZW and CT: conceptualization. CT: methodology. JY, LD, and CT: validation. YH: formal analysis and writing – original draft preparation. GC and DL: investigation. JY and CT: resources. YL and LY: data curation. YH and CT: writing, review, and editing. ZW: supervision, project administration, and funding acquisition. All authors have read and agreed to the published version of the manuscript.

## Conflict of Interest

YH, JY, LD, YL, LY, GC, CT, and ZW were employed by company Wens Foodstuff Group Co., Ltd. The remaining author declares that the research was conducted in the absence of any commercial or financial relationships that could be construed as a potential conflict of interest.

## Publisher’s Note

All claims expressed in this article are solely those of the authors and do not necessarily represent those of their affiliated organizations, or those of the publisher, the editors and the reviewers. Any product that may be evaluated in this article, or claim that may be made by its manufacturer, is not guaranteed or endorsed by the publisher.

## Footnotes

1
https://morningchores.com/large-white-pig

2
https://asia.ensembl.org/

3
https://metascape.org/
