# Supplementary material for: Genome-Wide Association Study for Body Length, Body Height, and Total Teat Number in Large White Pigs
Source: Front Genet. 2021 Aug 2;12:650370. doi: 10.3389/fgene.2021.650370 (PMC8366400; doi:10.3389/fgene.2021.650370)
Supplement: Supplementary file 1 [file Data_Sheet_1.docx]

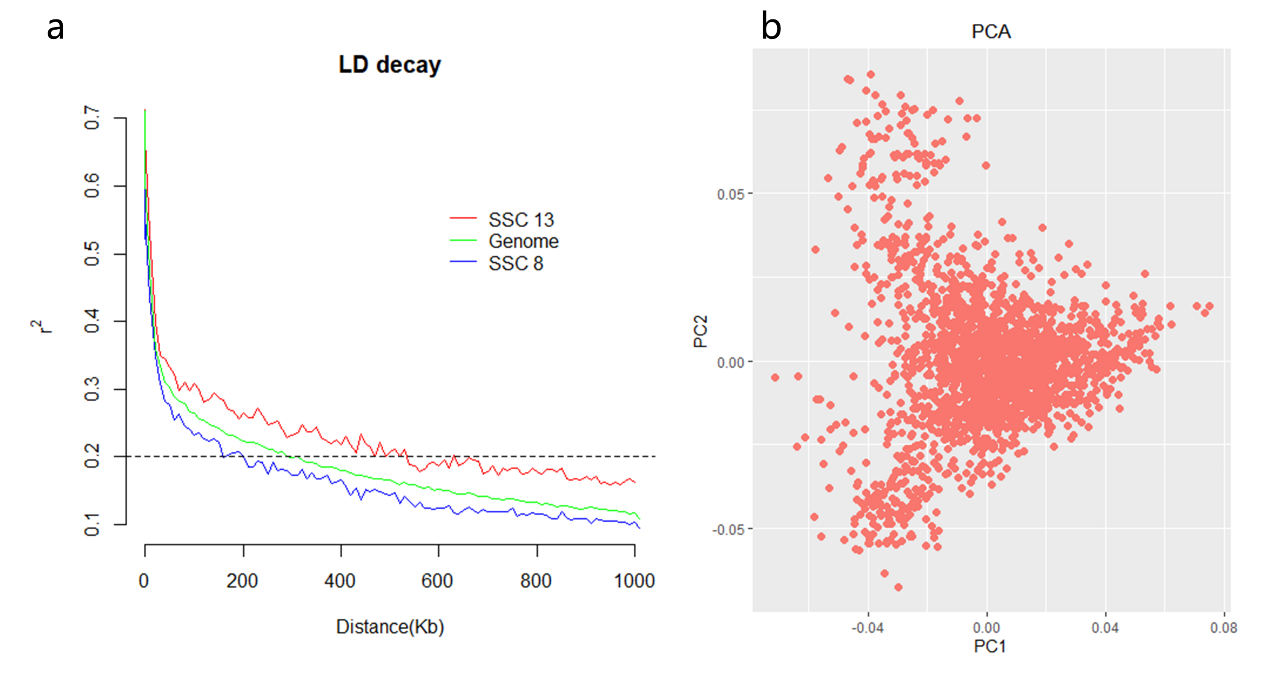


Supplementary Figure. 1 Genetic architecture of the Large White pig population. The figure on the left shows the principal component 1 and 2 distribution in the Large White pig population. The figure on the right shows the extent of linkage disequilibrium (LD), in which the LD on chromosomes 13 (SSC 13) and 8 (SSC 8) represent the highest and lowest levels across the whole genome, respectively.


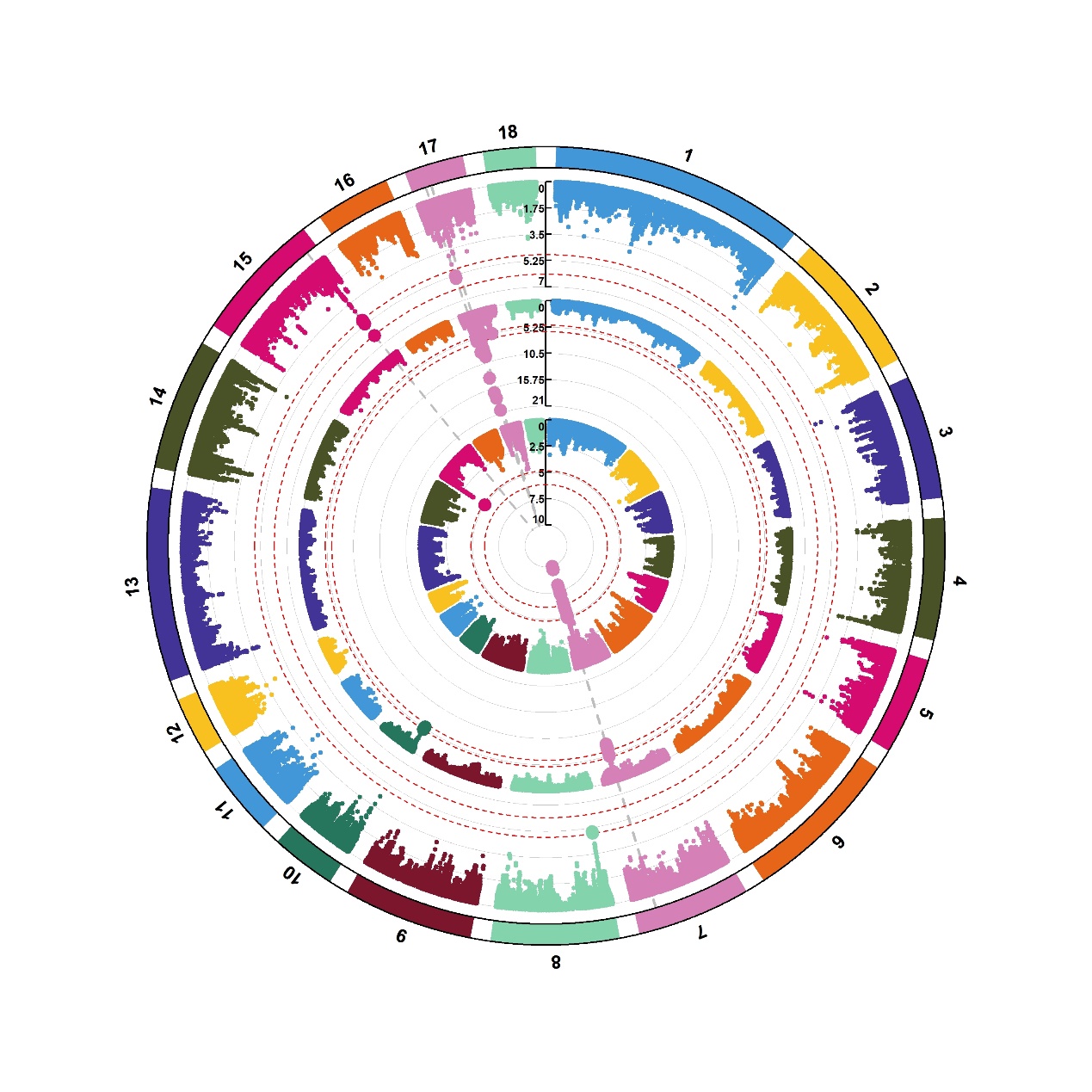


Supplementary Figure. 2 Circular Manhattan plots of the body length, body height and total number teats. Total teat number, body length and body height trait form inside out.


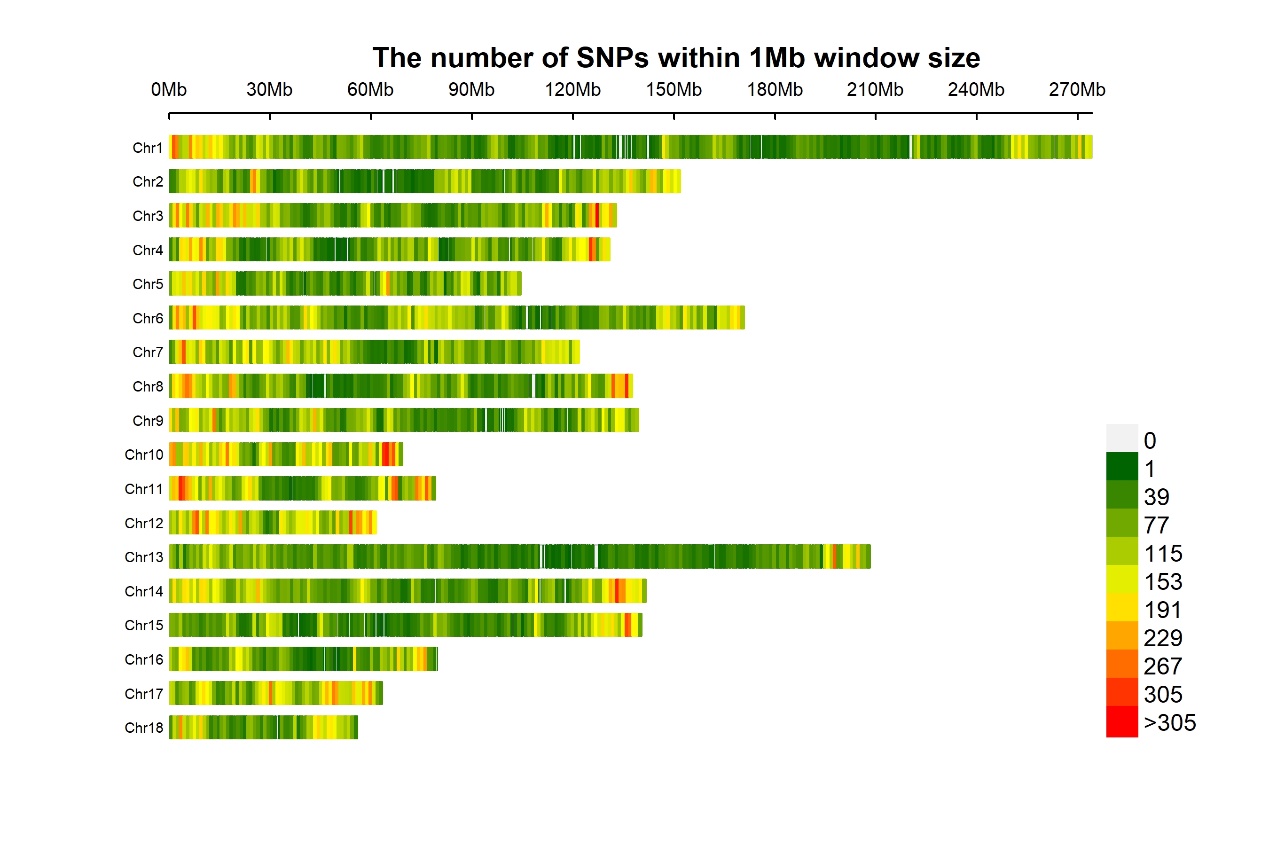


Supplementary Figure. 3 The location of SNP markers in chromosome. The SNP in these maps is from homologous pairing area in autosomes, these maps calculated the number of SNP markers within 1M. As the number of SNPs increases, the color changes from green to red and gradually deepens.


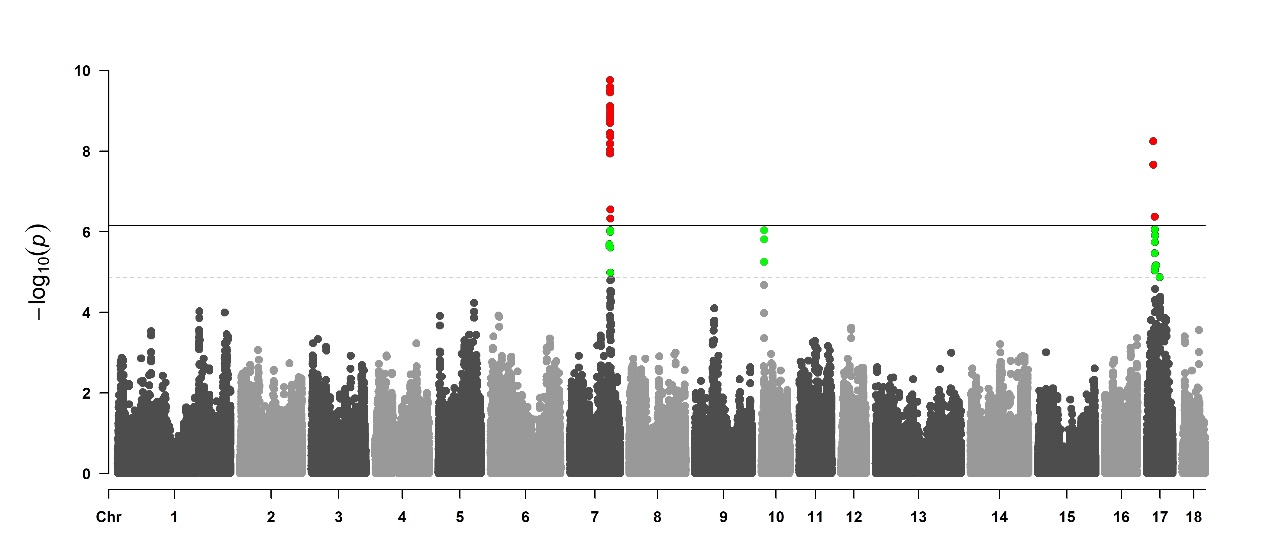


a

b


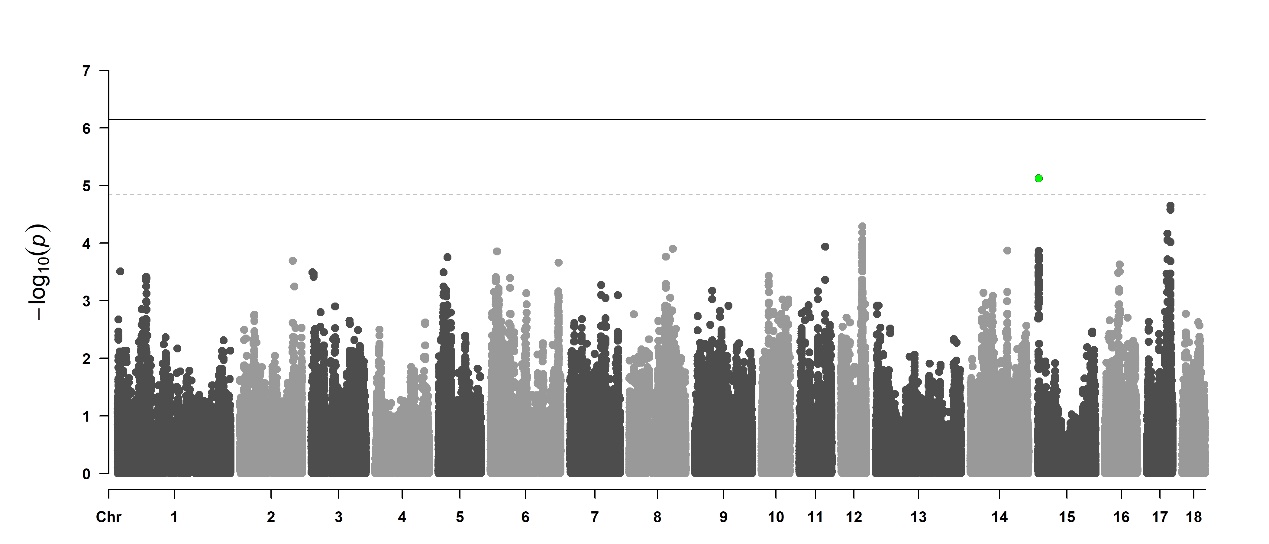


Supplementary Figure. 4 Take the point with the lowest P value as the covariant, Manhattan plots of the (a) body length and (b) total number teats. The X axis shows SNPs across chromosomes SSC1 to SSC18, and the Y axis represents the -log10(P). The top lines indicate the genome-wide significant thresholds (P < 7.03E-7) and the bottom line indicate the suggestive genome-wide significant thresholds (P < 1.41E-5).

Supplementary table. 1 Summary of detected QTLs for body length, body height and total teat number in Large White population

| ^1^Traits | ^2^SSC | ^3^SNP ID | ^4^Ps | ^5^Allele | ^6^MAF | P-value | Nearest gene | ^7^Distance(bp) | variance |
| --- | --- | --- | --- | --- | --- | --- | --- | --- | --- |
| BL | 7 | S7_97480599 | 97480599 | C/T | 0.30 | 1.78E-07 | LIN52 | 76740 | 0.51% |
|  | 7 | S7_97578184 | 97578184 | A/C | 0.29 | 9.39E-08 | ABCD4 | within | 1.17% |
|  | 7 | S7_97595288 | 97595288 | T/C | 0.29 | 1.27E-07 | ABCD4 | 9609 | 1.09% |
|  | 7 | S7_97595964 | 97595964 | G/A | 0.29 | 3.08E-07 | ABCD4 | 10285 | 1.88% |
|  | 7 | S7_97595973 | 97595973 | C/T | 0.29 | 1.50E-07 | ABCD4 | 10294 | 1.92% |
|  | 7 | S7_97690890 | 97690890 | A/G | 0.30 | 1.29E-07 | VRTN | 66617 | 0.10% |
|  | 7 | S7_97876972 | 97876972 | T/G | 0.30 | 4.62E-08 | LTBP2 | 24470 | 1.03% |
|  | 7 | S7_97877077 | 97877077 | G/A | 0.30 | 4.83E-08 | AREL1 | within | 0.95% |
|  | 7 | S7_97877153 | 97877153 | C/T | 0.30 | 2.95E-08 | AREL1 | within | 0.92% |
|  | 7 | S7_97877163 | 97877163 | G/C | 0.30 | 2.95E-08 | AREL1 | within | 0.92% |
|  | 7 | S7_97877233 | 97877233 | T/A | 0.30 | 2.47E-08 | AREL1 | within | 1.31% |
|  | 7 | S7_97877555 | 97877555 | C/T | 0.30 | 2.89E-08 | AREL1 | within | 1.21% |
|  | 7 | S7_97909776 | 97909776 | A/G | 0.30 | 7.25E-08 | AREL1 | within | 0.54% |
|  | 7 | S7_97919385 | 97919385 | C/G | 0.31 | 8.25E-08 | AREL1 | within | 0.45% |
|  | 7 | S7_97941907 | 97941907 | A/G | 0.31 | 3.83E-07 | FCF1 | within | 0.66% |
|  | 7 | S7_97941918 | 97941918 | A/G | 0.31 | 3.83E-07 | FCF1 | within | 0.66% |
|  | 7 | S7_97953008 | 97953008 | G/A | 0.31 | 5.18E-07 | FCF1 | within | 1.24% |
|  | 7 | S7_97953112 | 97953112 | T/C | 0.31 | 3.25E-07 | FCF1 | within | 1.39% |
|  | 7 | S7_97953147 | 97953147 | G/A | 0.31 | 1.37E-07 | FCF1 | within | 1.12% |
|  | 7 | S7_97953153 | 97953153 | A/G | 0.31 | 1.37E-07 | FCF1 | within | 1.12% |
|  | 7 | S7_98075416 | 98075416 | T/C | 0.30 | 7.49E-08 | PROX2 | 594 | 0.76% |
|  | 7 | S7_98075503 | 98075503 | C/T | 0.30 | 5.31E-08 | PROX2 | 681 | 0.78% |
|  | 7 | S7_98075512 | 98075512 | C/T | 0.30 | 5.31E-08 | PROX2 | 690 | 0.78% |
|  | 7 | S7_98111374 | 98111374 | G/C | 0.30 | 2.58E-07 | DLST | within | 0.89% |
|  | 7 | S7_98181824 | 98181824 | A/G | 0.16 | 4.36E-06 | EIF2B2 | -77359 | 0.30% |
|  | 7 | S7_98181957 | 98181957 | T/C | 0.16 | 7.59E-06 | EIF2B2 | -77226 | 0.54% |
|  | 7 | S7_98181958 | 98181958 | T/G | 0.16 | 7.59E-06 | EIF2B2 | -77225 | 0.54% |
|  | 10 | S10_6166929 | 6166929 | T/G | 0.06 | 1.38E-05 | usherin | -231916 | 0.32% |
|  | 10 | S10_6308100 | 6308100 | C/G | 0.06 | 2.99E-06 | usherin | -90745 | 0.72% |
|  | 10 | S10_6308305 | 6308305 | T/C | 0.07 | 4.93E-06 | usherin | -90540 | 0.13% |
|  | 17 | S17_15391920 | 15391920 | C/T | 0.16 | 3.33E-06 | ENSSSCG00000043546 | 59765 | 4.10% |
|  | 17 | S17_15404566 | 15404566 | G/T | 0.16 | 1.11E-05 | ENSSSCG00000043546 | 72411 | 4.10% |
|  | 17 | S17_15404571 | 15404571 | G/A | 0.16 | 1.11E-05 | ENSSSCG00000043546 | 72416 | 4.10% |
|  | 17 | S17_15414073 | 15414073 | T/C | 0.17 | 5.12E-06 | ENSSSCG00000043546 | 81918 | 3.98% |
|  | 17 | S17_15419600 | 15419600 | A/C | 0.15 | 1.05E-06 | ENSSSCG00000043546 | 87445 | 5.36% |
|  | 17 | S17_15419636 | 15419636 | G/A | 0.15 | 1.05E-06 | ENSSSCG00000043546 | 87481 | 5.36% |
|  | 17 | S17_15667418 | 15667418 | G/A | 0.42 | 1.23E-18 | BMP2 | -82417 | 6.94% |
|  | 17 | S17_15667503 | 15667503 | T/C | 0.42 | 1.23E-18 | BMP2 | -82332 | 6.94% |
|  | 17 | S17_15667902 | 15667902 | G/T | 0.43 | 1.97E-17 | BMP2 | -81933 | 7.55% |
|  | 17 | S17_15752736 | 15752736 | A/C | 0.18 | 6.09E-09 | BMP2 | within | 1.35% |
|  | 17 | S17_15781225 | 15781225 | C/A | 0.46 | 2.21E-14 | BMP2 | 20010 | 8.58% |
|  | 17 | S17_15781294 | 15781294 | A/G | 0.35 | 3.89E-21 | BMP2 | 20079 | 9.09% |
|  | 17 | S17_15781304 | 15781304 | T/C | 0.35 | 3.89E-21 | BMP2 | 20089 | 9.09% |
|  | 17 | S17_15843507 | 15843507 | T/A | 0.37 | 1.23E-05 | BMP2 | 82292 | 2.04% |
|  | 17 | S17_16988065 | 16988065 | G/A | 0.16 | 1.87E-06 | TMX4 | 106735 | 3.60% |
|  | 17 | S17_17054218 | 17054218 | T/A | 0.26 | 8.38E-06 | PLCB1 | within | 5.53% |
|  | 17 | S17_17054291 | 17054291 | A/G | 0.26 | 8.38E-06 | PLCB1 | within | 5.53% |
|  | 17 | S17_17140192 | 17140192 | A/T | 0.44 | 1.65E-06 | PLCB1 | within | 4.16% |
|  | 17 | S17_17140215 | 17140215 | A/G | 0.44 | 1.65E-06 | PLCB1 | within | 4.16% |
|  | 17 | S17_17140220 | 17140220 | G/A | 0.44 | 1.65E-06 | PLCB1 | within | 4.16% |
|  | 17 | S17_17140243 | 17140243 | T/G | 0.44 | 1.65E-06 | PLCB1 | within | 4.16% |
|  | 17 | S17_17216081 | 17216081 | G/A | 0.46 | 1.52E-06 | PLCB1 | within | 3.20% |
|  | 17 | S17_17216085 | 17216085 | G/A | 0.46 | 1.52E-06 | PLCB1 | within | 3.20% |
|  | 17 | S17_17216255 | 17216255 | C/G | 0.48 | 2.19E-06 | PLCB1 | within | 4.22% |
|  | 17 | S17_17262451 | 17262451 | A/G | 0.48 | 8.03E-07 | PLCB1 | within | 4.92% |
|  | 17 | S17_17265499 | 17265499 | T/A | 0.45 | 2.37E-06 | PLCB1 | within | 3.38% |
|  | 17 | S17_17266366 | 17266366 | C/T | 0.46 | 1.12E-05 | PLCB1 | within | 3.86% |
|  | 17 | S17_19318621 | 19318621 | A/T | 0.28 | 1.24E-05 | MKKS | -22356 | 2.45% |
|  | 17 | S17_22047913 | 22047913 | A/G | 0.48 | 1.68E-07 | TASP1 | within | 3.52% |
|  | 17 | S17_22048489 | 22048489 | C/T | 0.47 | 3.83E-06 | TASP1 | within | 3.36% |
|  | 17 | S17_22050846 | 22050846 | A/G | 0.17 | 8.57E-09 | TASP1 | within | 6.78% |
|  | 17 | S17_22230821 | 22230821 | A/G | 0.09 | 1.80E-06 | TASP1 | within | 1.48% |
|  | 17 | S17_22321096 | 22321096 | G/A | 0.16 | 4.55E-10 | TASP1 | within | 7.30% |
|  | 17 | S17_22321126 | 22321126 | C/A | 0.16 | 6.54E-10 | TASP1 | within | 7.46% |
|  | 17 | S17_22321201 | 22321201 | G/A | 0.16 | 4.55E-10 | TASP1 | within | 7.30% |
|  | 17 | S17_22321229 | 22321229 | C/T | 0.16 | 4.55E-10 | TASP1 | within | 7.30% |
|  | 17 | S17_22324664 | 22324664 | G/A | 0.17 | 2.83E-08 | TASP1 | within | 7.69% |
|  | 17 | S17_22324683 | 22324683 | T/G | 0.17 | 2.83E-08 | TASP1 | within | 7.69% |
|  | 17 | S17_22327859 | 22327859 | A/T | 0.09 | 1.33E-06 | TASP1 | within | 0.03% |
|  | 17 | S17_22327893 | 22327893 | T/C | 0.09 | 1.33E-06 | TASP1 | within | 0.03% |
|  | 17 | S17_22358074 | 22358074 | G/A | 0.09 | 6.66E-07 | TASP1 | 598 | 1.99% |
|  | 17 | S17_23133919 | 23133919 | T/C | 0.08 | 9.22E-07 | MACROD2 | within | 1.57% |
|  | 17 | S17_24155355 | 24155355 | A/C | 0.09 | 3.76E-06 | MACROD2 | within | 2.51% |
|  | 17 | S17_29687412 | 29687412 | A/G | 0.14 | 1.19E-05 | FOXA2 | -286588 | 1.61% |
|  | 17 | S17_47181100 | 47181100 | T/C | 0.30 | 6.55E-06 | RIMS4 | within | 5.34% |
|  | 17 | S17_47196996 | 47196996 | C/T | 0.30 | 5.97E-06 | RIMS4 | within | 5.20% |
| BH | 8 | S8_10099289 | 10099289 | T/C | 0.09 | 1.35E-05 | CPEB2 | -372849 | 0.42% |
|  | 8 | S8_10141092 | 10141092 | C/T | 0.09 | 1.11E-05 | CPEB2 | -331046 | 0.46% |
|  | 8 | S8_10141093 | 10141093 | A/G | 0.09 | 1.11E-05 | CPEB2 | -331045 | 0.46% |
|  | 8 | S8_10141101 | 10141101 | G/C | 0.09 | 1.11E-05 | CPEB2 | -331037 | 0.46% |
|  | 15 | S15_127806749 | 127806749 | A/G | 0.18 | 6.44E-06 | ENSSSCG00000044486 | -220920 | 6.07% |
|  | 15 | S15_127806772 | 127806772 | G/A | 0.18 | 6.44E-06 | ENSSSCG00000044486 | -220897 | 6.07% |
|  | 15 | S15_127817316 | 127817316 | C/T | 0.18 | 7.57E-06 | ENSSSCG00000044486 | -210353 | 5.33% |
|  | 15 | S15_127848108 | 127848108 | C/T | 0.18 | 6.37E-07 | ENSSSCG00000044486 | -179561 | 4.78% |
|  | 15 | S15_128229216 | 128229216 | G/A | 0.28 | 1.24E-05 | ENSSSCG00000044486 | 196561 | 3.73% |
|  | 15 | S15_128229245 | 128229245 | G/A | 0.28 | 1.21E-05 | ENSSSCG00000044486 | 196590 | 3.84% |
|  | 17 | S17_15781225 | 15781225 | C/A | 0.46 | 5.09E-06 | BMP2 | 20010 | 7.43% |
|  | 17 | S17_15781294 | 15781294 | A/G | 0.35 | 3.36E-06 | BMP2 | 20079 | 9.57% |
|  | 17 | S17_15781304 | 15781304 | T/C | 0.35 | 3.36E-06 | BMP2 | 20089 | 9.57% |
| TTN | 7 | S7_97150250 | 97150250 | G/A | 0.45 | 1.13E-05 | MIDEAS | 27284 | 3.97% |
|  | 7 | S7_97480599 | 97480599 | C/T | 0.30 | 7.83E-09 | LIN53 | 76740 | 8.20% |
|  | 7 | S7_97578184 | 97578184 | A/C | 0.29 | 2.30E-10 | ABCD4 | within | 9.17% |
|  | 7 | S7_97595288 | 97595288 | T/C | 0.29 | 1.71E-10 | ABCD4 | 9609 | 8.84% |
|  | 7 | S7_97595964 | 97595964 | G/A | 0.29 | 2.42E-10 | ABCD4 | 10285 | 9.06% |
|  | 7 | S7_97595973 | 97595973 | C/T | 0.29 | 1.11E-10 | ABCD4 | 10294 | 8.92% |
|  | 7 | S7_97690890 | 97690890 | A/G | 0.30 | 1.27E-08 | VRTN | 66617 | 7.59% |
|  | 7 | S7_97876972 | 97876972 | T/G | 0.30 | 8.17E-08 | LTBP2 | 24470 | 7.22% |
|  | 7 | S7_97877077 | 97877077 | G/A | 0.30 | 4.16E-08 | AREL1 | within | 7.06% |
|  | 7 | S7_97877153 | 97877153 | C/T | 0.30 | 3.37E-08 | AREL1 | within | 6.87% |
|  | 7 | S7_97877163 | 97877163 | G/C | 0.30 | 3.37E-08 | AREL1 | within | 6.87% |
|  | 7 | S7_97877233 | 97877233 | T/A | 0.30 | 4.48E-08 | AREL1 | within | 0.55% |
|  | 7 | S7_97877555 | 97877555 | C/T | 0.30 | 5.90E-08 | AREL1 | within | 6.64% |
|  | 7 | S7_97909776 | 97909776 | A/G | 0.30 | 3.68E-07 | AREL1 | within | 5.85% |
|  | 7 | S7_97919385 | 97919385 | C/G | 0.31 | 1.54E-06 | AREL1 | within | 6.03% |
|  | 7 | S7_97941907 | 97941907 | A/G | 0.31 | 2.57E-06 | FCF1 | within | 6.97% |
|  | 7 | S7_97941918 | 97941918 | A/G | 0.31 | 2.57E-06 | FCF1 | within | 6.97% |
|  | 7 | S7_97953008 | 97953008 | G/A | 0.31 | 3.08E-06 | FCF1 | within | 6.68% |
|  | 7 | S7_97953112 | 97953112 | T/C | 0.31 | 1.93E-06 | FCF1 | within | 6.77% |
|  | 7 | S7_97953147 | 97953147 | G/A | 0.31 | 1.95E-06 | FCF1 | within | 6.62% |
|  | 7 | S7_97953153 | 97953153 | A/G | 0.31 | 1.95E-06 | FCF1 | within | 6.62% |
|  | 7 | S7_98075416 | 98075416 | T/C | 0.30 | 5.20E-06 | PROX2 | 594 | 6.27% |
|  | 7 | S7_98075503 | 98075503 | C/T | 0.30 | 6.75E-06 | PROX2 | 681 | 6.42% |
|  | 7 | S7_98075512 | 98075512 | C/T | 0.30 | 6.75E-06 | PROX2 | 690 | 6.42% |
|  | 7 | S7_98111374 | 98111374 | G/C | 0.30 | 1.36E-05 | DLST | within | 6.28% |
|  | 7 | S7_99041676 | 99041676 | A/G | 0.35 | 9.84E-07 | TTLL5 | within | 4.86% |
|  | 7 | S7_99041677 | 99041677 | C/T | 0.35 | 1.41E-06 | TTLL5 | within | 4.85% |
|  | 7 | S7_99072885 | 99072885 | A/T | 0.36 | 9.25E-06 | TTLL5 | within | 4.83% |
|  | 15 | S15_1750246 | 1750246 | T/G | 0.09 | 1.01E-05 | RND3 | 15827 | 6.21% |

^1^BL = body length, BH = body height, TTN = total teat number; ^2^SSC = Sus scrofa chromosome, ^3^SNP_ID = SNP’s name, ^4^Ps = SNP position in Ensemble, ^5^Allele = minor allele/major allele, ^6^MAF = minor allele frequency, ^7^Distance = the SNP located upstream/downstream of the nearest gene.

Supplementary table. 2 Windows that explained >0.5% of the additive genetic variance for body length, body height and total teat number in Large White population

| ^1^Traits | ^2^SSC | Range of SNP(Mb) | Number of SNP | Top variance of SNP positition | Candidate gene | Gen.var.expl.by SNP (%) |
| --- | --- | --- | --- | --- | --- | --- |
| BL | 7 | 97.20-97.87 | 36 | 97480599 | ZNF410, FAM161B, COQ6, ENTPD5,  BBOF1, ALDH6A1, LIN52, VSX2, ABCD4,  VRTN, SYNDIG1L, NPC2, ISCA2, LTBP2,  AREL1 | 0.66 |
| BL | 14 | 128.29-128.86 | 27 | 128640312 | FAM204A, PRLHR, CACUL1,  ENSSSCG00000050831 | 0.55 |
| BL | 17 | 16.89-17.27 | 34 | 17054147 | TMX4, PLCB1, ENSSSCG00000045119 | 0.75 |
| BH | 14 | 128.29-131.15 | 122 | 131067719 | FAM204A, PRLHR, CACUL1,  ENSSSCG00000050831, NANOS1, EIF3A,  ENSSSCG00000050381, DENND10, SFXN4,  PRDX3, GRK5, RGS10, TIAL1, BAG3,  INPP5F, MCMBP, SEC23IP, PLPP4, WDR11,  ENSSSCG00000043365, ENSCG00000041462 | 1 |
| TNN | 6 | 14.96-15.02 | 14 | 14963493 | haptoglobin, ENSSSCG00000002749,  DNX3B, PMFBP1, TXNL4B, | 0.61 |
| TNN | 7 | 97.18-97.88 | 43 | 97297122 | ENSSSCG00000002351, ZNF410, FAM161B,  COQ6, ENTPD5, BBOF1, ALDH6A1, LIN52,  VSX2, ABCD4, VRTN, ATNDIG1L, NPC2,  ISCA2, LTBP2, AREL1 | 0.86 |

^1^BL = body length, BH = body height, TTN = total teat number; ^2^SSC = Sus scrofa chromosom.

Supplementary table. 3 Clusters with body length, body height and total teat number representative enriched terms

| Trait^1^ | GO | Description | Count^2^ | %^3^ | Log10(P) |
| --- | --- | --- | --- | --- | --- |
| BL | GO:0045444 | fat cell differentiation | 4 | 13.79 | -4 |
|  | GO:0051186 | cofactor metabolic process | 5 | 17.24 | -3.45 |
|  | GO:0007610 | behavior | 4 | 13.79 | -2.47 |
|  | GO:0044782 | cilium organization | 3 | 10.34 | -2.03 |
| BH | GO:0032386 | regulation of intracellular transport | 3 | 16.67 | -2.57 |
|  | GO:0042326 | negative regulation of phosphorylation | 3 | 16.67 | -2.46 |
|  | GO:0016311 | dephosphorylation | 3 | 16.67 | -2.44 |
|  | GO:0003006 | developmental process involved in reproduction | 3 | 16.67 | -2.01 |
| TTN | GO:0051186 | cofactor metabolic process | 5 | 22.73 | -4.05 |
|  | GO:0044282 | small molecule catabolic process | 4 | 18.18 | -3.4 |

^1^BL = body length, BH = body height, TTN = total teat number; ^2^Count = the number of genes in the user-provided lists with membership in the given ontology term. ^3^% = the percentage of all of the provided genes that.
